# Association of Geomagnetic Disturbances and Suicide Attempts in Taiwan, 1997–2013: A Cross-Sectional Study

**DOI:** 10.3390/ijerph17041154

**Published:** 2020-02-12

**Authors:** Tsutomu Nishimura, I-Ju Tsai, Hiroyuki Yamauchi, Eiji Nakatani, Masanori Fukushima, Chung Y. Hsu

**Affiliations:** 1 Translational Research Center for Medical Innovation, Kobe 650-0047, Japan; nakatani.eiji.int@gmail.com (E.N.); mfukushi@tri-kobe.org (M.F.); 2 Institute for Advancement of Clinical and Translational Science (iACT), Graduate School of Medicine, Kyoto University, Kyoto 606-8507, Japan; 3 Management Office for Health Data, China Medical University Hospital, Taichung 404, Taiwan; hunch0815@hotmail.com; 4 College of Medicine, China Medical University, Taichung 404, Taiwan; 5 Earthquake Prediction Research Center, Tokyo 103-0014, Japan; hiroyuki.yamauchi19@gmail.com; 6 Division of Statistical Analysis, Research Support Center, Shizuoka General Hospital, Shizuoka 420-8527, Japan; 7 Graduate Institute of Biomedical Sciences, China Medical University, Taichung 404, Taiwan; hsucy63141@gmail.com

**Keywords:** electromagnetic field, geomagnetic field, geomagnetic storm, suicide

## Abstract

Background: A previous study in Japan found that monthly mean K index values were related to the monthly number of male, but not female, suicides. Correlations between geomagnetic disturbances and suicide/depression have also been reported in countries such as Canada, South Africa, Finland, Australia, Russia, and Japan. We have previously shown that stronger geomagnetism is linked to a higher standardized mortality ratio for suicide. To date, however, no published studies have reported the correlation between geomagnetic disturbances and suicide attempts in Taiwan. Methods: Data on the monthly number of suicide attempts in Taiwan from January 1997 to December 2013 were obtained. We performed a multivariable analysis, with the number of suicide attempts as the response variable and monthly Kp10 index, F10.7 index, sulfur dioxide, carbon monoxide, ozone, fine particulate matter (PM2.5), temperature, humidity, unemployment rate, and cosmic rays as the explanatory variables. Results: The multivariable analysis showed that Kp10 index, temperature, humidity, unemployment rate, and cosmic rays were associated with the number of male suicide attempts and that Kp10 index, F10.7 index, carbon monoxide, temperature, humidity, and unemployment rate were associated with the number of female suicide attempts. Conclusion: This is the first article reporting statistically significant relationships between the monthly number of male and female suicide attempts and the monthly mean Kp10 value in Taiwan.

## 1. Introduction

Worldwide, there were 804,000 deaths from suicide in 2012, and the global age-standardized suicide rate is estimated to be 11.4 (15.0 male and 8.0 female) per 100,000 population [1]. Previous suicide attempts, psychiatric disorders, abuse of alcohol and other substances, employment status, financial loss, despair, chronic pain, chronic illness, and family history of suicide have been reported as risk factors for suicide [1]. Environmental factors such as air pollution [2,3], electromagnetic fields [4,5], and season [6,7,8] have also been reported as suicide risk factors.

Epidemiological studies have reported the possibility of an electromagnetic field at electric utility sites or a power line affecting depression and suicide [4,5,9]. In previous research, correlation between geomagnetic disturbances and suicide/depression has been reported in countries such as Canada, South Africa, Finland, Australia, Russia, and Japan [8,10,11,12,13,14,15]. In our previous work, we have also shown that stronger geomagnetism is linked to a higher standardized mortality ratio for suicide [16]. However, we did not include air pollution data in our previous studies [10,16], and air pollution has previously been reported to be associated with suicide [2,3] and depression [17,18]. In this study, we included air pollution data in the analyses. Furthermore, no studies have yet reported a correlation between geomagnetic disturbances and suicide attempts in Taiwan. Taiwan and Japan have similar cultures. In addition, global warming has progressed, and the current climate in Taiwan may represent the future climate in Japan. 

In this study, we examined the correlations of geomagnetic disturbances, as the independent variables of interest, with suicide attempts in Taiwan after adjusting for other explanatory variables that have been reported to be correlated with suicide.

## 2. Materials and Methods 

### 2.1. Study Participants and Medical Records

To identify suicide attempts (International Classification of Diseases, Ninth Revision, Clinical Modification codes E950–E959), we linked two datasets from the National Health Insurance Research Database in Taiwan (http://nhird.nhri.org.tw/date_01_en.html) [19], i.e., in-patient expenditures and the registry for beneficiaries, using unique encrypted personal identifiers. The datasets contain in-patient claims and demographic data from 1997 to 2013 on the 23 million beneficiaries in the Taiwan National Health Insurance program, which covers over 99% of the entire population. We calculated the monthly number of suicide attempts from 1997 to 2013. We counted patients with more than one record of suicide attempt in the same month as a single case for that month.

### 2.2. Air Pollution Data

In this study, we also investigated whether suicide attempts were associated with air pollution variables such as sulfur dioxide (SO_2_), carbon monoxide (CO), ozone (O_3_), particulate matter ≤ 10 (PM_10_) or ≤ 2.5 (PM_2.5_) micrometers in diameter, nitrogen oxides (NO_x_), nitric oxide (NO), nitrogen dioxide (NO_2_), total hydrocarbons (THC), non-methane hydrocarbons (NMHC), and methane (CH_4_).

Data on daily air quality variables, including temperature (°C), relative humidity (%), and concentrations of SO_2_ (ppb), CO (ppm), O_3_ (ppm), PM_10_ (μg/m^3^), PM_2.5_ (μg/m^3^), NO_x_ (ppb), NO (ppb), NO_2_ (ppb), THC (ppm), NMHC (ppm), and CH_4_ (ppm), from 1997 to 2013 were obtained from the Taiwan Environmental Protection Administration (EPA) website (https://taqm.epa.gov.tw/taqm/en/YearlyDataDownload.aspx) [20]. There are 76 EPA air monitoring stations in Taiwan. Daily values of temperature, relative humidity, and concentrations of each pollutant from all stations were aggregated to determine monthly patterns. In a previous study in Japan, we included air pressure and day length in the analysis, but these two variables are available in Taiwan only after 2009, so were not included.

### 2.3. Unemployment Rate

Monthly unemployment rates from 1997 to 2013 were downloaded from the website of the Directorate General of Budget, Accounting, and Statistics of the Executive Yuan (https://www.dgbas.gov.tw/mp.asp?mp=1) [21].

### 2.4. Geomagnetic Field and Space-Related Data

The definition of K-index was described by Menviene et. al., ‘The K indices are based upon the range in the irregular variations, measured in the two horizontal geomagnetic components after eliminating the so-called non-K variations; the vertical component Z is not considered because Z transient variations may be dominated by internal induction effects.’ [22]. *Kp10* is an indicator of disturbances in the Earth’s magnetic field. The F10.7 index is an indicator of solar activity. Solar wind speed is referred to as bulk speed.

Data on the geomagnetic field and space-related variables were extracted from the National Aeronautics and Space Administration (NASA)/Goddard Space Flight Center (GSFC) OMNI dataset through OMNIWeb (https://omniweb.gsfc.nasa.gov/form/dx1.html) [23], and cosmic ray data were extracted from the website of the Institute of Cosmophysical Research and Radio Wave Propagation, Far Eastern Branch of the Russian Academy of Sciences (http://cr0.izmiran.ru/mgdn/main.htm) [24]. The geomagnetic field and the space-related variables *Kp10* index, F10.7 index, and bulk speed were downloaded through the OMNIWeb interface. Monthly geomagnetic field and space-related variables were calculated from the daily data.

### 2.5. Procedure and Statistical Analysis

We performed this analysis using a cross-sectional design. The number of suicide attempts, air pollution variables, unemployment rate, and geomagnetic field and space-related variables were merged by year and month. Pearson correlation coefficients were calculated for all two-variable combinations of temperature, relative humidity, air pollutants, and magnetic field-related variables. Any pair of variables with an absolute value of Pearson’s correlation coefficient > 0.7 was considered highly correlated, and we selected variables that had lower correlations with each other as explanatory variables for linear regression analysis.

In the linear regression analysis, the response variable was the monthly number of suicide attempts, and the explanatory variables were *Kp10* index, F10.7 index, SO_2_, CO, O_3_, PM_2.5_, temperature, humidity, unemployment rate, and cosmic rays. To investigate the association between monthly number of suicide attempts and all factors used in the univariate analysis, we used the multivariable regression model [25]. All statistical analyses were performed using SAS software, Version 9.4 (SAS Institute Inc., Carey, NC, USA).

### 2.6. Ethics Approval and Consent to Participate

In the National Health Insurance Research Database in Taiwan, patients’ personal information is encrypted to protect individuals’ privacy. Researchers are provided with anonymous identification numbers associated with relevant claims information, including sex, date of birth, medical services received, and prescriptions. Patient consent is not required to access the National Health Insurance Research Database. This study was judged to fulfill the conditions for exemption by the institutional review board of China Medical University (CMUH104-REC2-115-CR2), which also specifically waived the consent requirement.

In Japan, according to the “Ethical Guidelines for Medical and Health Research Involving Human Subjects,” the guidelines do not apply to research utilizing only specimens and information that have already been anonymized and that cannot be linked to individuals. No ethical review was necessary.

## 3. Results

### 3.1. Correlation of Air Pollutants with Geomagnetic Field and Space-Related Variables

Based on the Pearson correlation coefficients, we selected the following explanatory variables: *Kp10* index, F10.7 index, SO_2_, CO, O_3_, PM_2.5_, temperature, humidity, unemployment rate, and cosmic rays (Table 1 and Table 2).

### 3.2. Linear Regression Analysis for Monthly Number of Suicide Attempts

The monthly number of male suicide attempts ranged from 70 to 266, whereas the monthly number of female suicide attempts ranged from 98 to 308. Table 3 shows the results of the univariate regression analysis for male and female suicide attempts. All the variables except for O_3_ and PM_2.5_ were significantly associated with the number of male suicide attempts. *Kp10* index, F10.7 index, CO, temperature, and cosmic rays were associated with the number of female suicide attempts.

Table 4 presents the results of the multivariable analysis. *Kp10* index, temperature, humidity, unemployment rate, and cosmic rays were associated with the number of male suicide attempts. *Kp10* index, F10.7 index, CO, temperature, humidity, and unemployment rate were associated with the number of female suicide attempts.

### 3.3. Changes in the Monthly Number of Male Suicide Attempts and Monthly Kp10 Values

**Figure 1:**
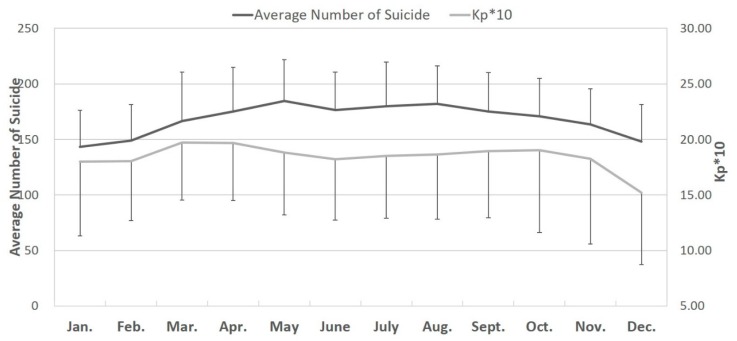
Changes in the monthly number of male suicide attempts and monthly *Kp10* values from January 1997 to December 2013.

**Figure 2:**
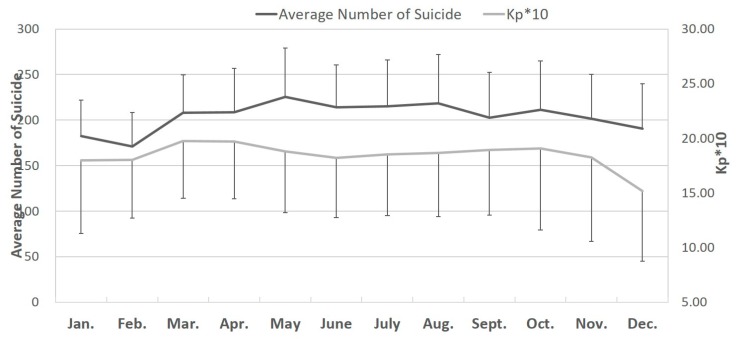
Changes in the monthly number of female suicide attempts and monthly *Kp10* values from January 1997 to December 2013.

Changes in the monthly number of male suicide attempts and monthly *Kp10* values are shown in Figure 1 and Figure 2. The peak in monthly number of male and female suicide attempts occurred in May (Figure 1 and Figure 2).

## 4. Discussion

In this study, we used data from Taiwan to confirm the reproducibility of our previous study in Japan. There was a relationship between the monthly number of male suicide attempts and the monthly mean *Kp10* value in Taiwan, as we had previously found to be the case in Japan.

In our previous study of men in Japan, monthly mean *K*-index value, number of sunspots, unemployment rate, air pressure, and humidity were related to the monthly number of suicides among males [10]. In the present study of men in Taiwan, monthly mean *Kp10* index, temperature, humidity, and unemployment rate were associated with the monthly number of male suicide attempts. The present findings for monthly mean *Kp10* index, humidity, and unemployment rate correspond with the findings of our previous study in Japan.

In our previous study of women in Japan, monthly mean air pressure and day length were associated with the monthly number of female suicides [10]. In the present study of women in Taiwan, monthly mean *Kp10* index, F10.7 index, CO, temperature, humidity, and unemployment rate were associated with the monthly number of female suicides. We were unable to include air pressure and day length in the present analysis because of the lack of appropriate data for Taiwan. None of our findings on the factors relating to female suicide attempts correspond with the results of our previous study in Japan. However, the present study’s finding that monthly mean CO was associated with the monthly number of female suicide attempts does correspond with other previous reports showing the relationship between air pollution and suicide [2,3].

In previous studies in Japan, the Kp index was used [10], but because the Kp index in Taiwan was not available, the *Kp10* index was used in this study. *Kp10* is an indicator of global geomagnetic disturbances. Since geomagnetic disturbances are a global phenomenon, we consider it appropriate to have used the *Kp10* index in this study.

Regarding the mechanism by which geomagnetism affects human health, there have been many reports on melatonin reduction due to geomagnetic disturbances [26]. Other potential mechanisms may influence the human mind through opioid receptors, as described in previous papers [10]. 

As to the synchronization of geomagnetic storms and suicide attempts, i.e., either delayed or immediate effects of geomagnetic disturbances on suicide attempts, Kay reported ‘significant 36.2% increase in male hospital admissions with a diagnosis of depressed phase, manic-depressive illness in the second week following such storms compared with geomagnetically quiet control periods.’ [15]. 

In this study, temperature and humidity were added to the covariates. Rainfall, which would reflect the effects of typhoons and heavy rains, was not used due to the short time period of available data. However, in this study, humidity was added to the covariates, which may reflect the effects of typhoons and heavy rain. We are considering analyzing earthquakes in a future study.

In contrast to the finding that monthly mean CO was positively associated with the monthly number of female suicide attempts, the CO regression coefficient for male suicide attempts was sufficiently large and negative (Table 3). Because sex differences have been reported in the impact of PM_2.5_ on depression [17], similar sex differences may be present in the impact of CO. There also seem to be gender differences in response to other environmental factors such as F10.7 index and cosmic rays.

A previous study using data in Japan found no association between the average *K*-index value and the monthly average number of female suicides [10]. However, in the present study in Taiwan, the monthly mean *Kp10* value was related to the monthly number of both male and female suicide attempts. 

In previous work, we have shown that stronger geomagnetism is linked to a higher standardized mortality ratio for suicide [16]. There is a possibility that the influence of geomagnetism is smaller in a location in which geomagnetism is weaker. Moving to a location with weaker geomagnetism could be one solution.

Limitations of the present study are the complicated environmental and ecological factors influencing suicides, which meant that we could not deny the possibility that the seasonality of geomagnetic disturbances [27] and the seasonality of suicides [6,7,8] tend to synchronize with each other.

## 5. Conclusions

The multivariable analysis showed that *Kp10* index, temperature, humidity, unemployment rate, and cosmic rays were associated with the number of male suicide attempts and that *Kp10* index, F10.7 index, carbon monoxide, temperature, humidity, and unemployment rate were associated with the number of female suicide attempts.

These findings are the first to report a statistically significant relationship between the monthly number of male and female suicide attempts and the monthly mean *Kp10* value in Taiwan.

## Figures and Tables

**Table 1:** Correlation coefficients for air pollutants during the study period.

Covariate	SO_2_^a^	CO ^b^	O_3_^c^	PM_10_^d^	PM_2.5_^e^	NO_x_^f^	NO ^g^	NO_2_^h^	THC ^i^	NMHC ^j^	CH_4_^k^	Temperature	Humidity
SO_2_	1												
CO	0.68	1											
O_3_	−0.17	−0.26	1										
PM_10_	0.57	0.50	0.42	1									
PM_2.5_	0.55	0.63	0.25	**0.80**	1								
NO_x_	**0.77**	**0.92**	−0.26	0.63	0.69	1							
NO	**0.72**	**0.86**	−0.56	0.33	0.43	**0.92**	1						
NO_2_	**0.72**	**0.87**	−0.01	**0.78**	**0.80**	**0.96**	**0.77**	1					
THC	0.50	**0.85**	−0.21	0.39	0.63	**0.80**	**0.73**	**0.77**	1				
NMHC	0.61	**0.82**	−0.26	0.45	0.66	**0.83**	**0.77**	**0.79**	**0.94**	1			
CH_4_	0.42	**0.83**	−0.17	0.33	0.58	**0.74**	0.67	**0.72**	**0.98**	**0.85**	1		
Temperature	−0.45	−0.50	−0.10	**−0.77**	−0.66	−0.67	−0.46	**−0.75**	−0.35	−0.44	−0.29	1	
Humidity	0.22	0.64	−0.43	−0.04	0.02	0.54	0.63	0.41	0.58	0.49	0.59	−0.11	1

Bold typeface indicates a coefficient with more than 0.70 or less than −0.70. ^a^ sulfur dioxide (ppb), ^b^ carbon monoxide (ppm), ^c^ ozone (ppm), ^d^ PM: particulate matter_10_ (μg/m^3^), ^e^ PM: particulate matter_2.5_ (μg/m^3^), ^f^ nitrogen oxides (ppb), ^g^ nitric oxide (ppb), ^h^ nitrogen dioxide (ppb), ^i^ total hydrocarbons (ppm), ^j^ non-methane hydrocarbons (ppm), ^k^ methane (ppm).

**Table 2:** Correlation coefficients for geomagnetic field and space-related variables during the study period.

Covariate	*Kp10*	F10.7	Bulk Speed
*Kp10*	1		
F10.7	0.50	1	
Bulk speed	**0.81**	0.16	1

Bold typeface indicates a coefficient with more than 0.70 or less than −0.70.

**Table 3:** Results of the univariate linear regression analysis.

Covariate	Male			Female		
	Coefficient	SE ^a^	*p*-Value	Coefficient	SE	*p*-Value
*Kp10* index	2.45	0.40	<0.0001	4.66	0.47	<0.0001
F10.7 index, solar flux units	0.19	0.06	0.004	0.50	0.08	<0.0001
SO_2_^b^, ppb	−13.4	2.64	<0.0001	−5.30	3.65	0.149
CO ^c^, ppm	−58.3	16.8	0.001	48.5	22.4	0.031
O_3_^d^, ppm	0.91	0.47	0.052	−0.36	0.61	0.556
PM_2.5_^e^, μg/m^3^	−0.39	0.23	0.084	0.16	0.30	0.606
Temperature, °C	2.98	0.57	<0.0001	2.89	0.77	0.0002
Humidity, %	−1.21	0.46	0.009	0.73	0.60	0.228
Unemployment rate, %	11.2	2.36	<0.0001	2.53	4.28	0.555
Cosmic rays	−0.04	0.01	<0.0001	−0.05	0.01	<0.0001

Response variable: Monthly number of suicide attempts from 1997 to 2013 (N = 204 for male suicide attempts; N = 204 for female suicide attempts); ^a^ standard error, ^b^ sulfur dioxide, ^c^ carbon monoxide, ^d^ ozone, ^e^ particulate matter_2.5_.

**Table 4:** Results of the multivariable analysis.

Covariate	Male			Female		
	Coefficient	SE ^a^	*p*-Value	Coefficient	SE	*p*-Value
*Kp10* index	2.44	0.49	<0.0001	3.23	0.60	<0.0001
F10.7 index, solar flux units	0.004	0.09	0.960	0.33	0.11	0.002
SO_2_^b^, ppb	6.25	4.02	0.120	9.23	5.06	0.068
CO ^c^, ppm	−0.30	34.9	0.993	164	44.2	0.0002
O_3_^d^, ppm	0.58	0.42	0.171	0.08	0.53	0.882
PM_2.5_^e^, μg/m3	−0.48	0.33	0.152	−0.69	0.41	0.094
Temperature, °C	2.12	0.67	0.002	4.04	0.83	<0.0001
Humidity, %	−1.58	0.61	0.009	−2.68	0.77	0.001
Unemployment rate, %	10.2	2.35	<0.0001	22.8	4.78	<0.0001
Cosmic rays	−0.02	0.01	0.010	−0.01	0.01	0.325

Response variable: Monthly number of suicide attempts from 1997 to 2013 (N = 204 for male suicide attempts; N = 204 for female suicide attempts). Explanatory variables: Variables listed in Table 1. ^a^ standard error, ^b^ sulfur dioxide, ^c^ carbon monoxide, ^d^ ozone (ppm), ^e^ particulate matter_2.5_.

## References

[1] World Health Organization (2014). Preventing Suicide: A Global Imperative.

[2] Bakian A.V., Huber R.S., Coon H., Gray D., Wilson P., McMahon W.M., Renshaw P.F. (2015). Acute air pollution exposure and risk of suicide completion. Am. J. Epidemiol..

[3] Ng C.F.S., Stickley A., Konishi S., Watanabe C. (2016). Ambient air pollution and suicide in Tokyo, 2001–2011. J. Affect. Disord..

[4] Reichmanis M., Perry F.S., Marino A., Becker R. (1979). Relation between suicide and the electromagnetic field of overhead power lines. Physiol. Chem. Phys..

[5] Van Wijngaarden E., Savitz D., Kleckner R.C., Cai J., Loomis D. (2000). Exposure to electromagnetic fields and suicide among electric utility workers: A nested case-control study. West. J. Med..

[6] Chew K.S.Y., McCleary R. (1995). The spring peak in suicides—A cross-national analysis. Soc. Sci. Med..

[7] Heerlein A., Valeria C., Medina B. (2006). Seasonal variation in suicidal deaths in Chile: Its relationship to latitude. Psychopathology.

[8] Partonen T., Haukka J., Nevanlinna H., Lönnqvist J. (2004). Analysis of the seasonal pattern in suicide. J. Affect. Disord..

[9] Perry S., Pearl L. (1988). Power frequency magnetic field and illness in multi-storey blocks. Public Health.

[10] Tada H., Nishimura T., Nakatani E., Matsuda K., Teramukai S., Fukushima M. (2014). Association of geomagnetic disturbances and suicides in Japan, 1999–2010. Environ. Health Prev. Med..

[11] Gordon C., Berk M. (2003). The effect of geomagnetic storms on suicide. S. Afr. Psychiatry Rev..

[12] Berk M., Dodd S., Henry M. (2006). Do ambient electromagnetic fields affect behaviour? A demonstration of the relationship between geomagnetic storm activity and suicide. Bioelectromagnetics.

[13] Brahic C. (2008). Does the Earth’s magnetic field cause suicides?. NewScientist.

[14] Ganjavi O., Schell B., Cachon J.-C., Porporino F. (1985). Geophysical variables and behavior: XXIX. Impact of atmospheric conditions on occurrences of individual violence among Canadian penitentiary populations. Percept. Mot. Ski..

[15] Kay R.W. (1994). Geomagnetic storms: Association with incidence of depression as measured by hospital admission. Br. J. Psychiatry.

[16] Nishimura T., Tada H., Nakatani E., Matsuda K., Teramukai S., Fukushima M. (2014). Stronger geomagnetic fields may be a risk factor of male suicides. Psychiatry Clin. Neurosci..

[17] Wang F., Liu H., Liu J., Guo X., Yuan J., Hu Y., Wang J., Lu L., Li H. (2018). Ambient concentrations of particulate matter and hospitalization for depression in 26 Chinese cities: A case-crossover study. Environ. Int..

[18] Szyszkowicz M., Kousha T., Kingsbury M., Colman I. (2016). Air Pollution and Emergency Department Visits for Depression: A Multicity Case-Crossover Study. Environ. Health Insights.

[19] National Health Insurance Research Database in Taiwan (NHIRD). http://nhird.nhri.org.tw/date_01_en.html.

[20] Taiwan Environmental Protection Administration (EPA) Website. https://taqm.epa.gov.tw/taqm/en/YearlyDataDownload.aspx.

[21] Directorate General of Budget, Accounting, and Statistics of the Executive Yuan. https://knoema.com/atlas/sources/DGBAS?topic=CPI.

[22] Mandea M., Korte M. (2010). Geomagnetic Observations and Models.

[23] NASA/GSFC’s OMNI Data Set. https://omniweb.gsfc.nasa.gov/form/dx1.html.

[24] Institute of Cosmophysical Research and Radio Wave Propagation, Far Eastern Branch of the Russian Academy of Sciences. http://cr0.izmiran.ru/mgdn/main.htm.

[25] Harrell J.F.E. (2015). SpringerLink. Regression Modeling Strategies: With Applications to Linear Models, Logistic and Ordinal Regression, and Survival Analysis.

[26] Cherry N. (2002). Schumann resonances, a plausible biophysical mechanism for the human health effects of Solar/Geomagnetic activity. Nat. Hazards.

[27] Lyatsky W., Hamza A.M. (2001). Seasonal and diurnal variations of geomagnetic activity and their role in Space Weather forecast. Can. J. Phys..

